# Supplementary material for: Sirtuin5 protects colorectal cancer from DNA damage by keeping nucleotide availability
Source: Nat Commun. 2022 Oct 17;13:6121. doi: 10.1038/s41467-022-33903-8 (PMC9576705; doi:10.1038/s41467-022-33903-8)
Supplement: Supplementary file 1 — Supplementary Info [file 41467_2022_33903_MOESM1_ESM.pdf]

Sirtuin5 protects colorectal cancer from DNA damage by keeping nucleotide availability

Hao-Lian Wang[1,3], Yan Chen[1,3], Yun-Qian Wang[1,3], En-Wei Tao[1], Juan Tan[1], Qian-Qian Liu[1], Chun-Min Li[1], Xue-Mei Tong[2], Qin-Yan Gao[1], Jie Hong[1], Ying-Xuan Chen[1*] & Jing-Yuan Fang[1]

[1]State Key Laboratory for Oncogenes and Related Genes; Division of Gastroenterology and Hepatology, Renji Hospital, School of Medicine, Shanghai Jiao Tong University, Shanghai, China.

[2]Department of Biochemistry and Molecular Cell Biology, Shanghai Key Laboratory for Tumor Microenvironment and Inflammation, Key Laboratory of Cell Differentiation and Apoptosis of Chinese Ministry of Education, Shanghai Jiao Tong University School of Medicine, Shanghai, China.

[3]Hao-Lian Wang, Yan Chen and Yun-Qian Wang contributed equally to this work.

[*]Correspondence and requests for materials should be addressed to Y.-X.C. (email: yingxuanchen71@sjtu.edu.cn).

Supplementary information

Supplementary Figure 1

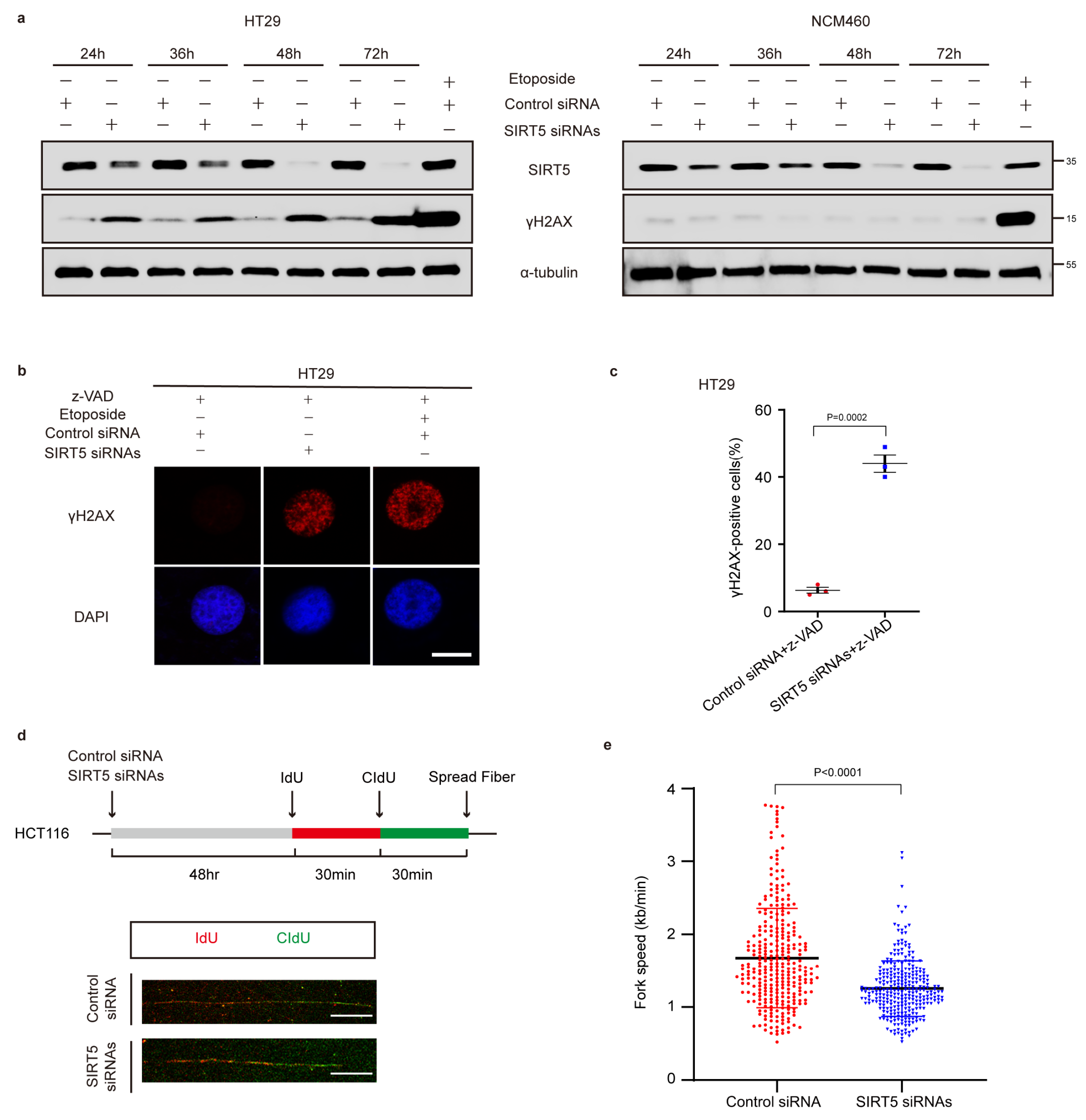

Supplementary Figure 1. (a) Western blotting to assess γH2AX (Ser139) levels in HT29 and NCM460 cells transfected with negative control (NC) or SIRT5 siRNAs for 24, 36, 48, and 72 h. (b, c) Representative images of γH2AX (Ser139) immunofluorescence staining (b) and quantification (c) in HT29 cells with SIRT5 knockdown. Etoposide served as a positive control. Scale bar, 5 μm. (n = 3 biologically independent experiments). (d, e) Control and SIRT5-depleted HCT116 cells were pulsed using a

labeling scheme. Representative fiber images for indicated samples are shown (d). CldU and IdU lengths were measured and converted to kilo bases (1 μm = 2.59 Kb), and Fork speed was plotted as histograms (e). Scale bar, 5 μm. 300 fibers were recorded from three biologically independent samples. Values indicate mean ± standard deviation (SD) in (c) and mean ± standard error of the mean (SEM) in (e). Statistical significance was calculated using two-tailed unpaired *t*-test.

Supplementary Figure 2

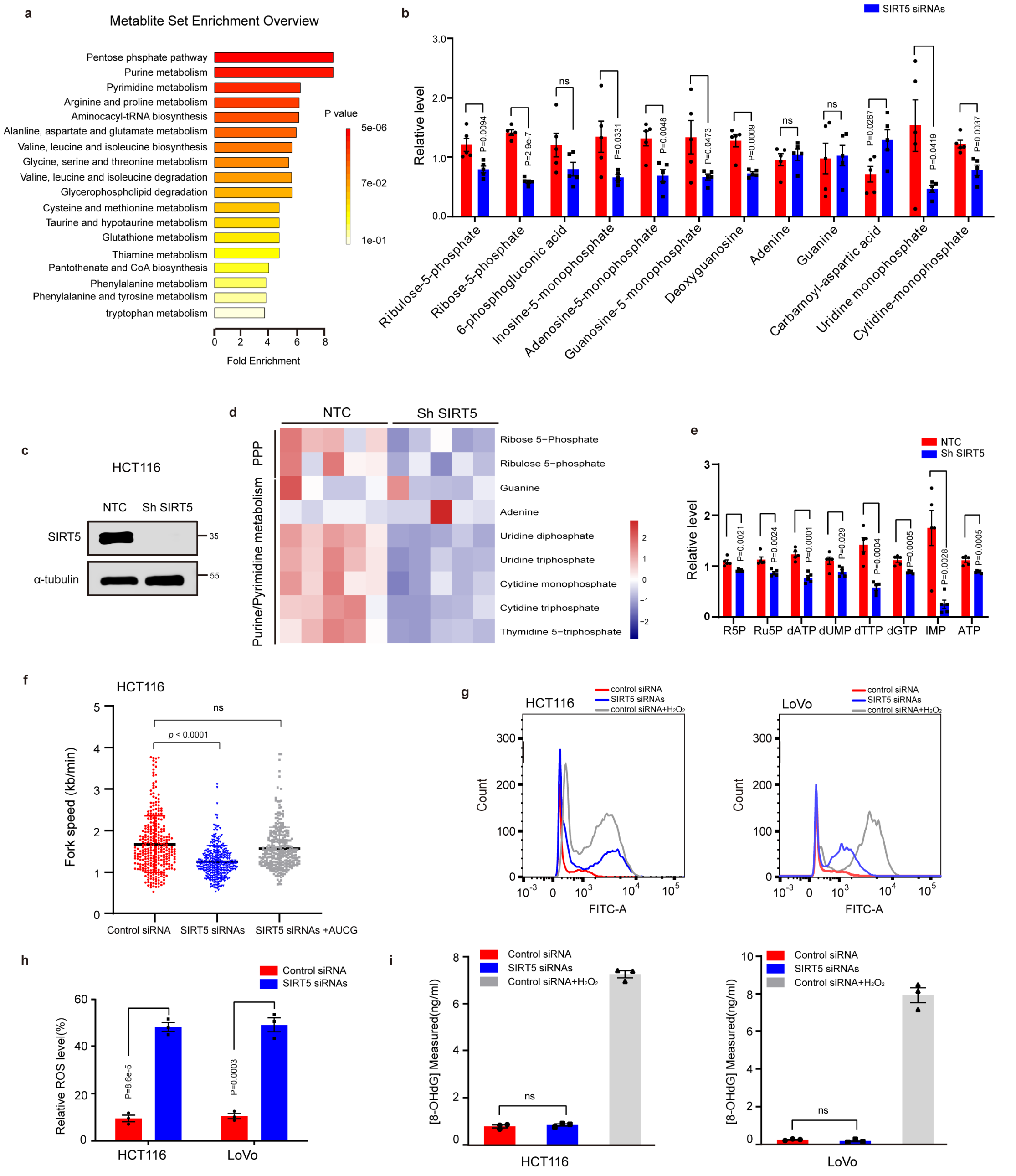

Supplementary Figure 2. (a) Metabolite set enrichment analysis of differentially abundant metabolites in HCT116 cells (NC or SIRT5 siRNAs) was performed using gas chromatography-mass spectrometry (GC–MS). (b) Quantitative analysis of the PPP metabolites and nucleotide intermediates in HCT116 cells transfected with NC or SIRT5 siRNAs. Values represent the mean ± SEM. (n = 5 biologically independent experiments). (c) HCT116 cells were transduced with lentiviral particles carrying

SIRT5 short hairpin RNAs (shRNAs) or non-target control (NTC) shRNA, and infected cells were selected using puromycin. SIRT5 depletion was confirmed in stably SIRT5-transduced cells. (d) Heatmap showing significantly differentially expressed metabolites in the PPP and purine/pyrimidine metabolism pathway after stable knockdown of SIRT5 in HCT116 cells. (n = 5 biologically independent experiments). (e) Targeted metabolomics analysis of R5P and nucleotides in HCT116 cells with stable deletion of SIRT5. Values represent mean ± SEM. (n = 5 biologically independent experiments). (f) Fork speed was performed in SIRT5-silenced HCT116 cells after supplementation of exogenous nucleosides. 300 fibers were recorded from three biologically independent samples. (g, h) ROS levels were detected using the DCFDA probe after SIRT5 depletion in HCT116 and LoVo cells. Data in g is quantified (h). (n = 3 biologically independent experiments). (i) 8-OH-dG levels were detected by ELISA in NC or SIRT5 siRNAs-transfected HCT116 (left) and LoVo (right) cells. H_2O_2 served as a positive control. (n = 3 biologically independent experiments). Values in f, h, and i represent mean ± SEM. Statistical significance was calculated by two-tailed unpaired *t*-test（b, e, h) or one-way ANOVA corrected with Tukey's multiple comparisons test (f, i). ns, not significant. Source data are provided as a Source Data file.

Supplementary Figure 3

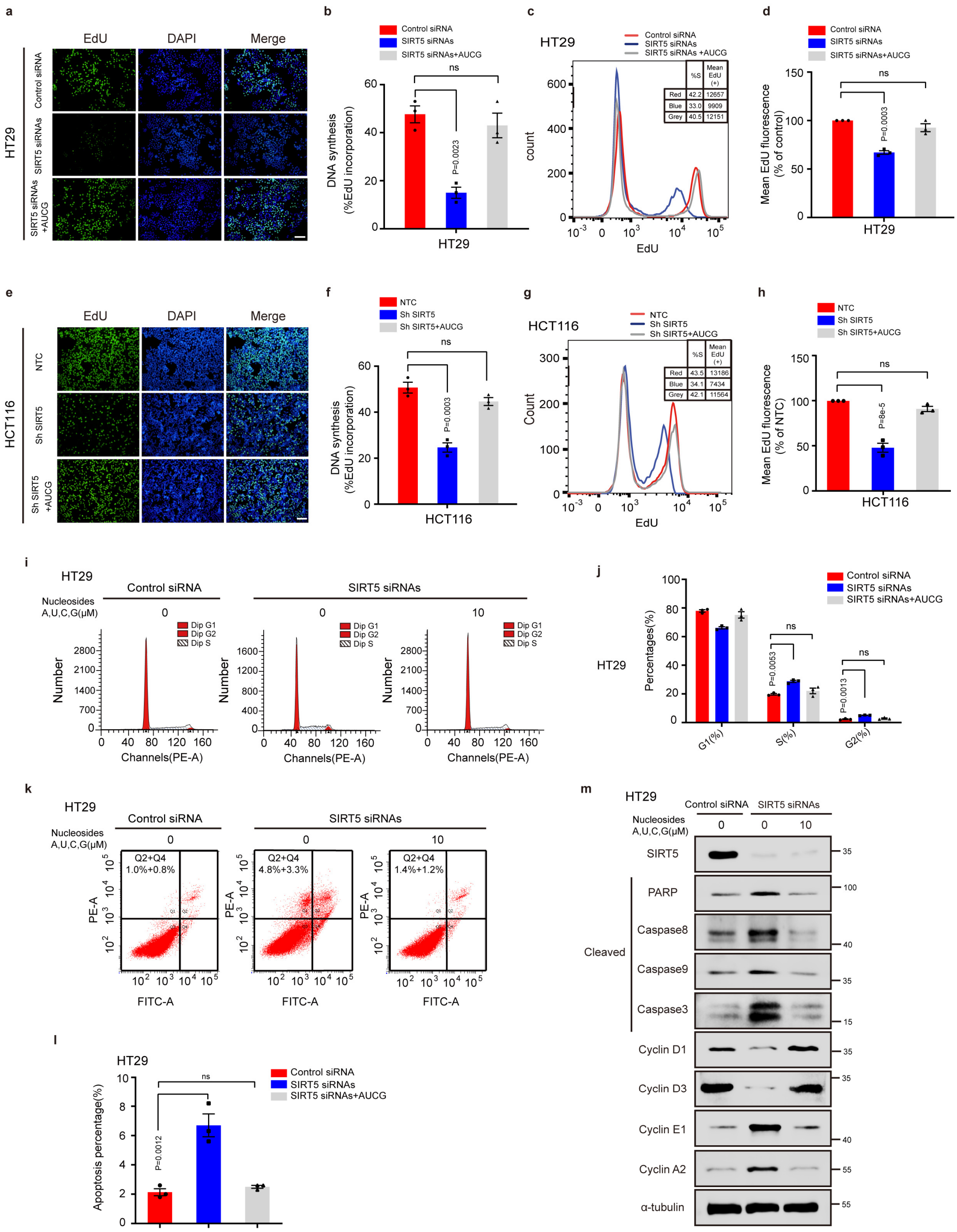

Supplementary Figure 3. (a–d) DNA synthesis was measured by immunofluorescent staining (a) and flow cytometry (c) in HT29 cells transfected with SIRT5 siRNAs after exogenous nucleoside supplementation. The data in a and c were quantified and analyzed in (b) and (d) respectively. Scale bars, 50 μm. (e–h) EdU assay involving

immunofluorescent staining (e) and flow cytometry (g) of HCT116 cells with stable SIRT5 knockdown after exogenous nucleoside supplementation. Scale bars indicate 50 μm. The data in e and g were quantified and analyzed in (f) and (h) respectively. (i–l) Flow cytometry was performed to detect changes in the cell cycle (i) and apoptosis (k) in SIRT5-deficient HT29 cells with exogenous nucleoside supplementation for 16 h. The data in i and k were quantified and analyzed in (j) and (l) respectively. (m) Western blotting analysis of apoptosis indicators (the cleaved caspase 8, caspase 9, caspase 3, and PARP) and cell cycle regulators in SIRT5-silenced HT29 cells after supplementation with nucleosides. Values in b, d, f, h, j, and l are mean ± SEM of three independent experiments. Statistical significance was calculated using one-way ANOVA with multiple comparisons corrected by Tukey's test. ns, not significant. Source data are provided as a Source Data file.

Supplementary Figure 4

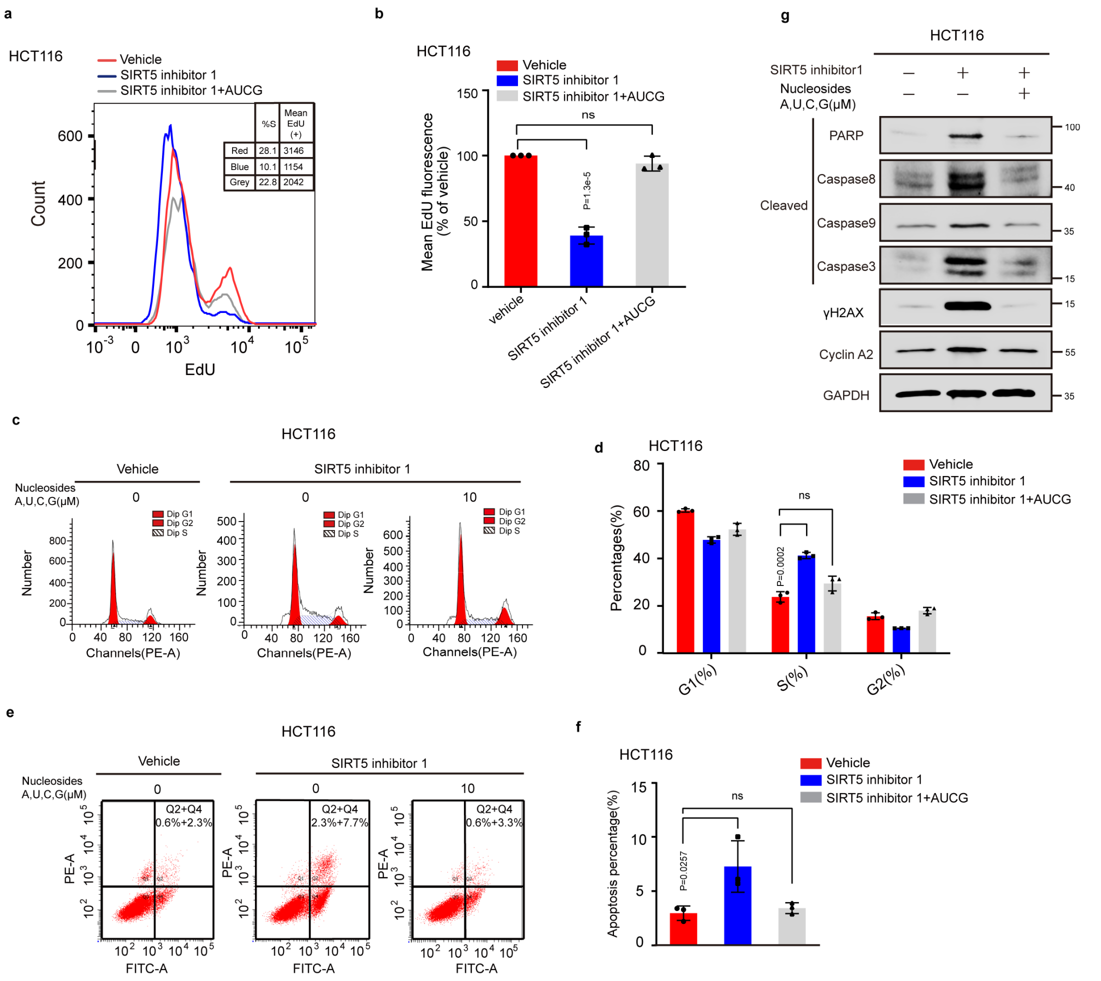

Supplementary Figure 4. (a, b) Cells were treated with 50 μM SIRT5 inhibitor 1 for 24 h, and then cultured with four nucleosides for 16 h. DNA synthesis was measured using EdU flow cytometry assay (a). Data in a were quantified (b), representing the mean fluorescence intensity of the EdU^{+} population. (c–f) Cell cycle transition (c) and apoptosis (e) were detected by flow cytometry analysis of HCT116 cells treated with 50 μM SIRT5 inhibitor 1 for 24 h in the presence/absence of nucleosides. The data in c and e were quantified and analyzed in (d) and (f) respectively. (g) Exogenous nucleoside supplementation rescued SIRT5 inhibitor 1-induced apoptosis and DNA damage indicators. Cells were treated with 50 μM SIRT5 inhibitor 1 for 24 h in the

presence/absence of the four nucleosides. Values in b, d, and f represent the mean ± SD of three independent experiments. One-way ANOVA with Tukey's multiple comparisons test was used. ns, not significant. Source data are provided as a Source Data file.

Supplementary Figure 5

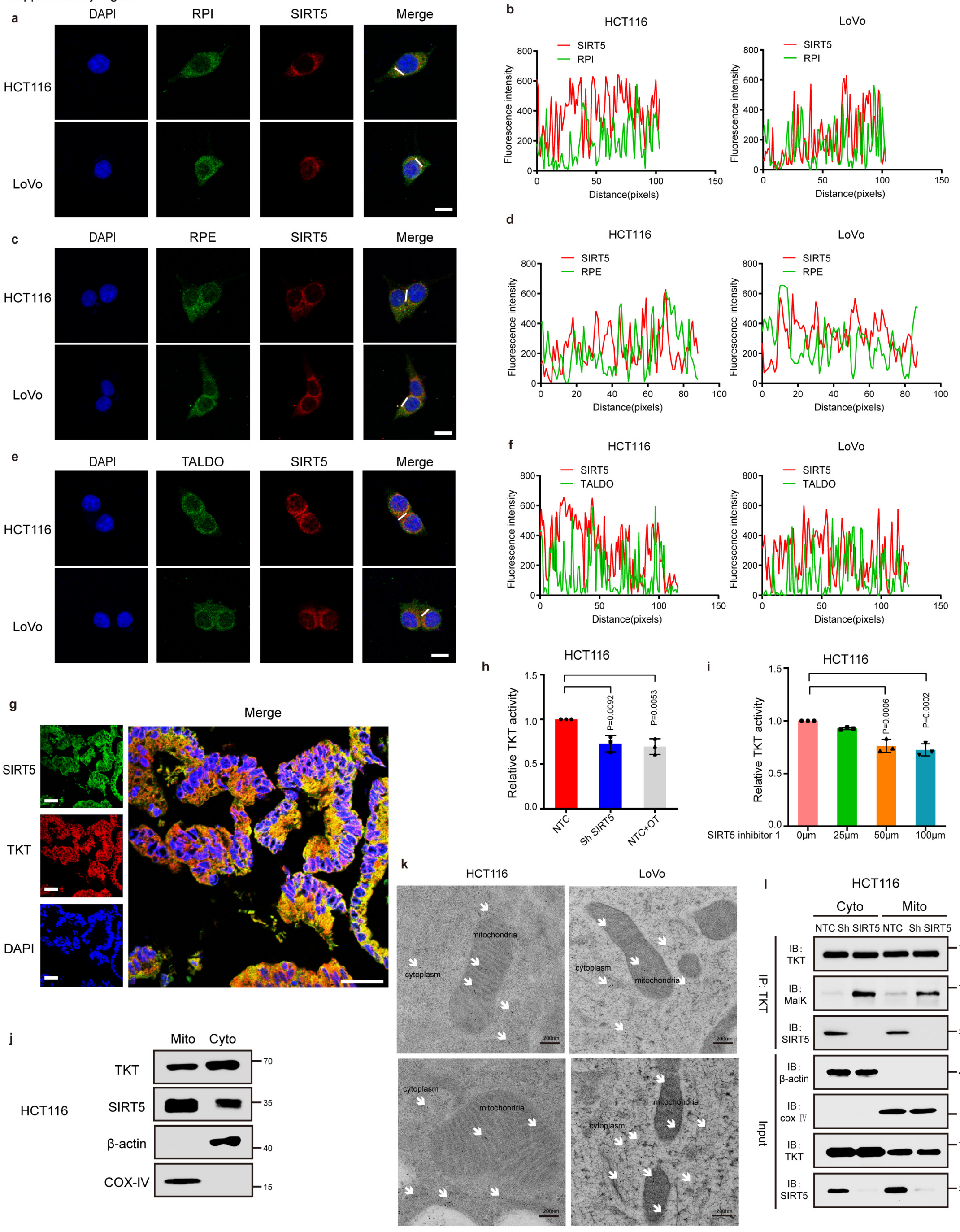

Supplementary Figure 5. (a–f) HCT116 and LoVo cells were immunostained for SIRT5 (in red) and RPI (a), RPE (c), and TALDO (e) (in green). Yellow in the merged magnified images (left) indicates co-localization. Scale bar, 10 μm. Fluorescence intensity of SIRT5 (red line) and RPI (b), RPE (d), and TALDO (f) (green line) traced along the white line in CRC cells using the line profiling function of ImageJ. (g)

Immunofluorescence analysis of TKT (red) and SIRT5 (green) expression in CRC tissues; yellow in the merged magnified images (left) indicates co-localization between TKT and SIRT5. Scale bar, 10 μm. (h) TKT enzyme activity was assessed on stable knockdown of SIRT5 in HCT116 cells. The TKT inhibitor oxythiamine (OT; 20 μM) served as a positive control. (i) HCT116 cells were treated with indicated concentrations of SIRT5 inhibitor 1 for 24 h. TKT activity was measured, as previously described. Data in h and i are presented as mean ± SD from three independent experiments. *P* values were calculated using one-way ANOVA with Tukey's multiple comparisons test. ns, not significant. Source data are provided as a Source Data file.

Supplementary Figure 6

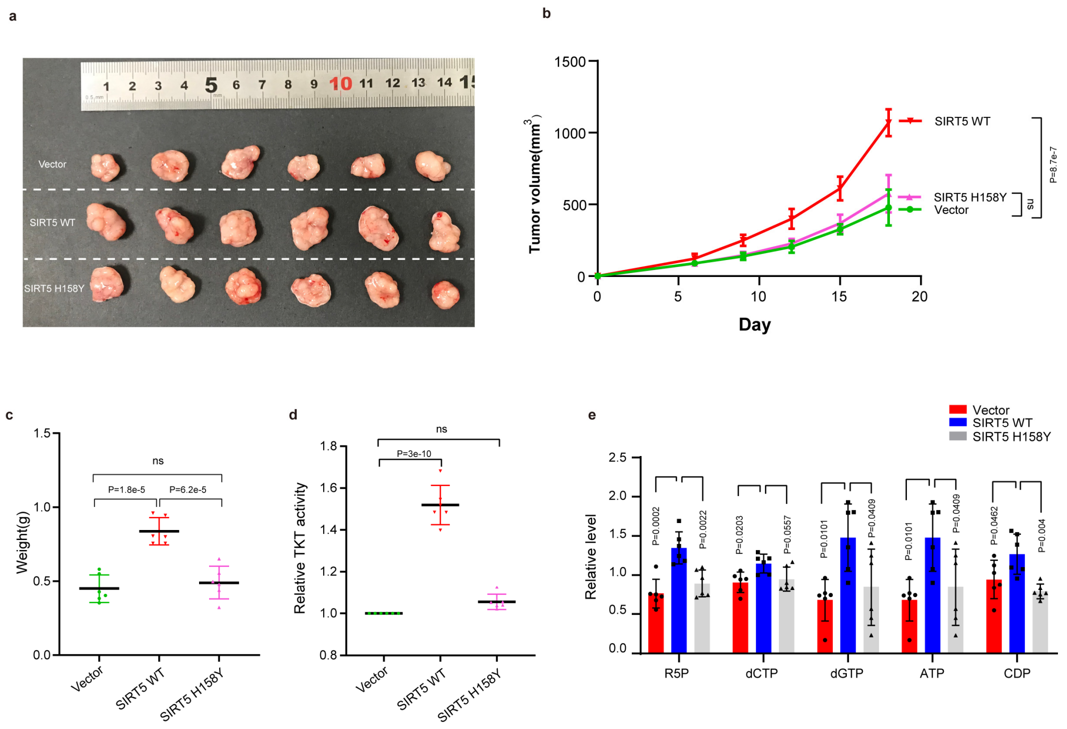

Supplementary Figure 6 (a) HCT116 cells stably expressing the control vector, SIRT5 WT, or SIRT5 H158Y were subcutaneously injected into nude mice (n = 6 mice per group). (b) Tumor volume was measured at indicated timepoints, and mean tumor volume was calculated (n = 6 mice per group). (c) At the end of the experiment, tumors from the three groups were weighed (n = 6 mice per group). (d) TKT activity in tumor lysates derived from subcutaneous xenograft tumors was measured (n = 6 mice per group). (e) Targeted metabolomics analysis of R5P and nucleotides in tumor lysates derived from subcutaneous xenograft tumors in nude mice with stable expression of the control vector, SIRT5 WT, or SIRT5 H158Y (n = 6 mice per group). Data in b, c, d, and e were shown as the mean ± SD. One-way ANOVA with Tukey's multiple comparisons test was used to assess significance. ns, not significant. Source data are provided as a Source Data file.

Supplementary Figure 7

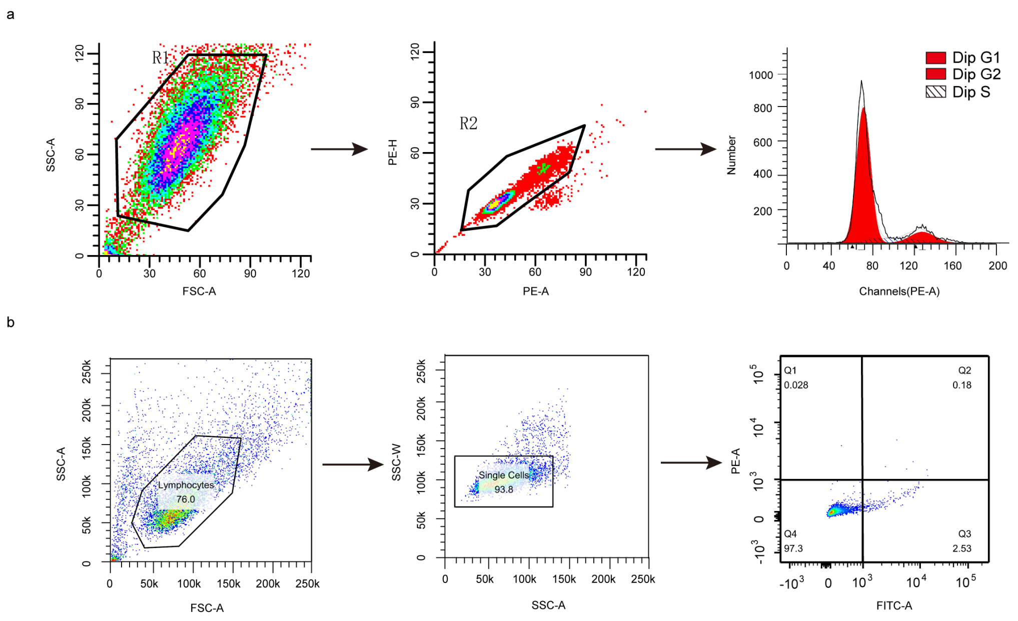

Supplementary Figure 7 (a–b) Scheme of the gating strategy used in flow cytometry assays for examining cell cycle (a) and apoptosis (b) of CRC cells under different treatment conditions.

Supplementary Table 1

Clinical and pathological information of 60 cases CRC patients.

Parameters	Number (%)
**Age**, y, mean ± SD	65.5 ± 9.6
**Gender**	
Male	37 (61.7)
Female	23 (38.3)
**Location**	
Colon	36(60.0)
Rectum	24(40.0)
**Grade**	
Ⅰ - Ⅱ	52(86.7)
Ⅲ	8(13.3)
